# Recent Advances in the Diagnosis and Treatment of Niemann-Pick Disease Type C in Children: A Guide to Early Diagnosis for the General Pediatrician

**DOI:** 10.1155/2015/816593

**Published:** 2015-02-16

**Authors:** Hanna Alobaidy

**Affiliations:** ^1^Department of Pediatrics, Tripoli University, Tripoli, Libya; ^2^Pediatrics Department, El Khadra Hospital, Tripoli, Libya

## Abstract

Niemann-Pick disease (NP-C) is a lysosomal storage disease in which impaired intracellular lipid transport leads to accumulation of cholesterol and glycosphingolipids in various neurovisceral tissues. It is an autosomal recessive disorder, caused by mutations in the *NPC1* or *NPC2* genes. The clinical spectrum is grouped by the age of onset and onset of neurological manifestation: pre/perinatal; early infantile; late infantile; and juvenile periods. The NP-C Suspicion Index (SI) screening tool was developed to identify suspected patients with this disease. It is especially good at recognizing the disease in patients older than four years of age. Biochemical tests involving genetic markers and Filipin staining of skin fibroblast are being employed to assist diagnosis. Therapy is mostly supportive and since 2009, the first specific therapy approved for use was Miglustat (Zavesca) aimed at stabilizing the rate of progression of neurological manifestation. The prognosis correlates with age at onset of neurological signs; patients with early onset form progress faster. The NP-C disease has heterogeneous neurovisceral manifestations. A SI is a screening tool that helps in diagnostic process. Filipin staining test is a specific biomarker diagnostic test. Miglustat is the first disease-specific therapy.

## 1. Introduction

Historical delineation: Albert Niemann first described the disease in 1914 in an infant with hepatosplenomegaly who died at 18 months after progressive neurological deterioration and was found to have large foam cells in liver and spleen [1]. In the late 1920s, Albert Niemann and Ludwig Pick worked together, the eponym “Niemann-Pick disease” has since been used to designate a heterogeneous group of lipid storage disorders, with common presentation of hepatosplenomegaly with or without neurological involvement [2]. In 1934, the stored lipid was identified by Klenk as sphingomyelin [1]. In 1958, Crocker and Farber reported that there was a wide variability in age of onset, clinical presentation, and level of sphingomyelin deposition in variable organs [2]. In 1966, Brady and colleagues identified the deficiency of sphingomyelinase as the cause of Niemann-Pick disease types A and B but not type C or D [1, 2]. Since the early 1980s, the Niemann-Pick disease has been divided in two entities, based on their metabolic defect: acid sphingomyelinase (ASM) deficiencies, including types A, B and intermediate forms, and lipid trafficking defect, corresponding to Niemann-Pick type C [3]. The term, Niemann-Pick disease type D, a genetic isolated from Nova Scotia, should no longer be used; it is shown to be a genetic variant of type C [2, 3]. Niemann-Pick disease type C (NP-C) is a rare autosomal recessive, lysosomal storage disease characterized by impaired intracellular lipid trafficking leading to accumulation of cholesterol and glycosphingolipids in various tissues. It is a progressive, irreversible disease caused by mutation in the genes* NPC1* (95% of cases) or* NPC2* (≈4% of cases). The minimal estimated incidence is 1 in 120,000 live births; this seems to be an underestimation, as NP-C is often misdiagnosed due to its heterogeneous presentation [2, 4]. The age of onset ranges from the prenatal period until late adult age. Most of the patients die between 10 and 25 years of age [5], NP-C is characterized by visceral, neurological, and psychiatric manifestation. The age at onset of neurological manifestations influences the prognosis, with earlier onset a greater mortality [5, 6]. There is no curative treatment for NP-C. Miglustat (Zavesca) is approved for the treatment of progressive neurological manifestations and works by inhibiting the glucosylceramide synthase enzyme, which is responsible for the first step in the synthesis of most glycosphingolipids [7]. Typically, it is challenging to diagnose patients with NP-C; they can present to different health care professionals including pediatricians. The aim of this review is to increase disease awareness of the timing of diagnosis by focusing on clinical presentation of NP-C and the diagnostic tool, where pediatrician may initiate evaluation of suspected patients, when team work is needed (metabolic specialist, neurologist, geneticist, and dietician); they should be referred to specialist center in order to start disease-specific treatment and to improve health-related quality of life (HR-QOL).

The molecular biology underlying NP-C pathophysiology: NP-C is associated with mutations of genes,* NPC1* and* NPC2*, with no primary defect in catabolic enzymes. These mutations cause severe impairment of intracellular lipid transport. When the NPC1 or the NPC2 protein is nonfunctional, the cellular trafficking of endocytosed LDL-derived cholesterol is impaired and leads to accumulation of unesterified cholesterol and other lipids in perinuclear lysosomes. The pattern of accumulating lipids is different in brain and nonneural organs. Accumulation of unesterified cholesterol, phospholipids, and glycosphingolipids in liver and spleen may result in organomegaly and liver dysfunction. On the other hand, neurons store only a minimal amount of cholesterol while levels of glycosylceramide, lactosylceramide, and GM2 and GM3 gangliosides are markedly increased in the brain [3, 5, 8].

## 2. Clinical Signs and Symptoms in NP-C

They are grouped by age of onset and onset of neurological manifestation.

*Pre/Perinatal*. The presence of perinatal hydrops fetalis or family history of a sibling with fetal ascites is a possible indicator of lysosomal storage disease. It is usually associated with progressive hepatosplenomegaly. The neonatal signs range from transient unexplained jaundice to severe cholestatic hepatopathy. Neurological involvement is not recognized during the neonatal period. Fatal lung infiltration with foam cells and a severe respiratory insufficiency is mostly restricted to patients having mutation in the* NPC2* gene [2, 5, 6].

*Early Infantile Period (Onset at 2 Months to 2 Years of Age).* The presentation of isolated splenomegaly or hepatosplenomegaly is the strongest visceral sign observed in most of patients with NP-C. Delay of developmental motor milestone and central hypotonia are the first neurologic symptoms with poor head control, abnormal upward saccades, and slow movement while transferring objects. Also present are the loss of acquired motor skills, spasticity, intention tremor, and hearing loss. Brain imagery may show leukodystrophy, white matter signal hyperintensity in T2, or brain atrophy which is the characteristic finding [2, 5, 6].

*Late Infantile Period (Onset at 2–6 Years of Age).* Hepatosplenomegaly, ataxia, clumsiness, and frequent falling are often present between ages of 3–5 years. Hearing loss, dysarthria with delayed speech and impaired pronunciation, and dysphagia are often present; focal or generalized seizures (sometimes fatal), cataplexy, and vertical supranuclear gaze palsy (VSGP) are usually present, while mental impairment becomes more marked [2, 4–6]. 

*Juvenile Presentation (Onset at 6–15 Years of Age).* This is the most common NP-C form. Organomegaly is not usually present. Detection of an isolated splenomegaly at this period is an initiatory sign of NP-C and should be carefully monitored. The most prominent are the neurological manifestations, characterized by learning disability and school failure with difficulties in writing and impaired attention and behavioral problems. Progressive ataxia in combination with dystonia of the hands and the face, dysarthria, dysphagia, and myoclonus are also present. Cataplexy is the episodes of sudden loss of muscle tone with sudden fall typically induced by laughter, VSGP results by impaired saccadic movements of the eyes in the vertical direction. About half of the patients develop seizures [2, 5, 6].

*Adolescent/Adult Presentation (*>*15 Years of Age).* Is classically show a neurological or psychiatric manifestations as ataxia, dystonia, dysarthria with variable cognitive dysfunction, psychiatric symptoms and dementia, while epilepsy, vertical gaze and splenomegaly are rare in adult NP-C patients [2, 5, 6].

## 3. Diagnostic Methods

The NP-C Suspicion Index (SI) tool should be used after the most common diseases have been ruled out (Table 1), an online tool is available at http://www.npc-si.com/. Its easy-to-use resource provides information to assist clinicians unfamiliar with NP-C in early identification of possible NP-C patients. The NP-C SI screening tool was recently developed by an international group of NP-C clinical experts; before using a tool, the data are necessary to prepare for each patient including age, gender, sign, and symptoms of NP-C within the three domains (visceral, neurological, and psychiatric) and patients first- or second-degree family history. The scoring system for the SI tool assigned points to individual NP-C manifestations within each domain and points in and for familial history. The sums of all points provide the total risk prediction score (RPS) (Table 2). Patients with a score of ≥70 indicate high suspicion of NP-C and should be immediately referred to an NP-C specialist center for testing, scores from 40 to 69 indicate moderate suspicion for NP-C and recommendation for further follow-up observation, and an NP-C referral center should be contacted for discussion. Scores <40 indicate a low probability for having NP-C. Neurological manifestations are the most common and sensitive symptoms of NP-C in SI tool, while visceral symptoms are the most specific, and psychiatric symptoms the least specific. Some symptoms of NP-C act as very strong indicators at different ages among patients suspected as having the disease but considered less sensitive in neonates and early infancy (e.g., VSGP is not usually seen at this age group); in addition, delayed developmental milestones considered an ancillary indicator in SI tool but could be considered a moderate indicator for neonates and young infants [4, 9].

For more accurate assessments, a new tool for use in the pediatrics population was developed [10], by an updated retrospective analysis of the original NP-C tool, and conducted by subanalysis of patients by age: infantile patients <4 years, juvenile patients 4–16 years, and adolescent patients >16 years of age, to identify the discriminatory power of the SI tool by age, redistribution of frequency of manifestations within and across domains, and RPS (indicator for the severity of the disease) categories <70, 70–150, and >150 points. Patients with low scores were most commonly seen in groups <4 years of age. High suspicion of NP-C with an RPS of >150 was characterized by increased presence of VSGP, splenomegaly, ataxia, and psychiatric manifestations. Two manifestations within the same domain greatly increased the scores. The estimated receiver operating characteristic (ROC) curve (sensitivity and specificity) area under the curve (AUC) values demonstrated strong discrimination power of a risk prediction score (RPS) for patients 4–16 years of age and patients >16 years of age, with AUC ROC of 0.981 and 0.964, respectively, and highest discriminatory power for the neurological manifestations, but were weak with ROC AUC value of 0.562 for patients (<4 years) with poor discriminatory power in all three domains, perhaps due to unrecognized neurological or psychiatric presentation in this age group [10]. A new NP-C diagnosis model, termed the 2/7 scores, is based on the presence of only 7 signs and symptoms and requires a combination of two signs to be present for a high suspicion of NP-C; this model proved highly sensitive and specific in patients >4 years of age [11]. There is still a need for a more reliable tool for use in pediatrics patients <4 years. A summary of the frequency of individual symptoms and signs and combinations by age group is presented in Table 3; the majority of manifestations in infantile patients appeared across the visceral and neurological domains and less in the psychiatric domain. The frequency of psychiatric manifestations significantly increased in juvenile and adolescent patients. Combined manifestations across the visceral/psychiatric, visceral/neurological, and neurological/psychiatric are increased in frequency in patients >4 years of age [10, 11]. Association by presence/absence of four leading NP-C manifestations (ataxia (moderate indicator), cognitive decline (strong indicator), psychosis (moderate indicator), and splenomegaly (strong indicator)) in patients >4 years is presented in Table 4; presence of one or more of these presentations most often indicated a strong suspicion of NP-C and patients should be referred for testing. On the other hand, other specific NP-C symptoms, such as VSGP, cataplexy, and epilepsy, were excluded as leading symptoms, due to their lower frequency, later onset, or difficulty of diagnosis in early infancy [10].

### 3.1. Initial Clinical Assessments for NP-C Patients Differ from Other Lysosomal Storage Diseases

Due to complex and unspecific clinical symptoms, the diagnosis is relatively difficult in cases lacking the typical symptoms. In order to properly diagnose, one should seek a combination of signs and symptoms for early detection such as the splenomegaly and VSGP or clumsiness or psychosis, or the ataxia with dysarthria/dysphagia and cognitive decline (Table 2).

### 3.2. A Full Medical History

Might include perinatal hydrops fetalis, onset of neonatal jaundice, organomegaly, history of splenectomy, neurological manifestations, abnormal muscle tone or posture, ataxia, dystonia, dysphagia, seizures, cataplexy, poor school performance, behavioral problems (agitation, hyperactivity, sleep disorders, visual hallucination or depression) and neuropsychiatric manifestations (psychosis, schizophrenia) where early warning signs [2, 5, 6, 12].

### 3.3. Clinical Examination

A comprehensive clinical examination should consider vital signs, body weight, height and head circumference, neurological examination, and ultrasound of liver and spleen. Clinical assessment of the disease progression can be evaluated by video record.

*Ocular motor abnormalities* include vertical supranuclear gaze palsy (VSGP) and saccadic eye movements (SEM) are present in most of patients and often the earliest sign seen in NP-C disease. It is often overlooked and most commonly detected from late infantile period onwards. The initial SEM deficit is in the vertical plane (VSEM) affecting patients' abilities to look downward, upward, or both. Similarly, patients with horizontal gaze (HSEM) may fall as they become unable to adjust their vision to climb stairs or other obstacles. They close their eyes when trying to look up and reopen having reached an upward position. Therefore, they may blink when asked to look upward. In older children, the defect appears more affecting the down gaze. This is manifested by tilting the head during writing or descending stairs. The ophthalmic assessments can be conducted based on video-recorded eye movements [2, 6, 13–15].

*Gelastic cataplexy* is the sudden loss of muscle tone, ranging from minor head drop to falls, to full collapse in response to humorous stimuli. Onset is as early as two years of age and is often confused with epilepsy. It is not associated with a loss of consciousness and can be characterized by normal electroencephalogram (EEG) [4, 6, 16].

*Hearing Loss.* A high frequency sensorineural hearing loss has been described in patients with late infantile onset form. This can be tested by audiograms or by brainstem evoked potentials [6].

*Cognitive impairment* may range from a delay in developmental milestones to mental retardation. The clinical course includes a loss of previously acquired abilities, delayed motor development (poor head control, delayed walking, clumsiness, frequent falls, and slow movements while walking, transferring, and picking up objects), and delay in social play, speech, and vision (lack of visual attention) [6]. Psychosis may be associated with visual hallucinations, catatonia, and fluctuating symptoms [6, 17].

*Dysphagia* is associated with dysfunction in brainstem, or the cortical areas in frontal lobe (the areas responsible initiating the swallowing action). It carries risk of recurrent aspiration pneumonia. The swallowing function evaluation can be assessed using food types of different consistencies or radiographic imaging video fluoroscopy [18, 19].

*Dysarthria* due to a lack of coordination of the motor-speech system results in irregular slurred speech [6].

*Ataxia* which is associated with early loss of Purkinje cells in the cerebellum may be associated with dystonia of the hands and the face. It can be assessed by the International Cooperative Ataxia Rating Scale [20] or provocation tests such as tandem gait, rapidly alternating movements, and finger-to-nose and heel-to-shin tests.

*Organomegaly.* Often observed is isolated unexplained splenomegaly (with or without hepatomegaly). The size of spleen does not correlate with neurological presentation or severity of the disease. Absence of splenomegaly does not exclude NP-C.

*Prolonged neonatal cholestatic jaundice* is defined as a conjugated bilirubin level of more than 1.2 mg/dL and more than 30% of total bilirubin for a period of more than 2 weeks. It is usually observed in patients with early or late infantile presentation. NP-C should be considered in the differential diagnosis with other causes of cholestatic jaundice [4, 9, 21].

## 4. Investigation

General nonspecific biochemical tests may give normal results: a full blood count and liver transaminases (aspartate aminotransferase AST and alanine aminotransferase ALT), serum bilirubin, and coagulation function are usually abnormal in patients with cholestatic jaundice or hepatosplenomegaly. A lipid profile correlated to severity of cholesterol trafficking abnormalities usually showing low HDL (high-density lipids) cholesterol [22]. Bone marrow aspiration could be useful in highly suspicious cases (prior to a specific biochemical test) which show the cholesterol-loaded foam cells (similar to cells in Gaucher disease).

*Specific Diagnostic Tests.* (1) The primary biomarker diagnostic test is the Filipin test. It is the most sensitive and specific test and should be conducted in specialized expert centers. It is a fibroblast culture of living cells from a skin biopsy. The fibroblasts are cultured in a low density lipid (LDL) enriched medium, fixed and stained with Filipin. Using fluorescence microscopic examination, one can see numerous cholesterol-filled perinuclear vesicles, in about 85% of cases [2, 4, 5, 23]. (2) When possible, DNA sequencing should be performed when the Filipin study is clearly positive. A mutation analysis can be vital in confirming the diagnosis in patients whose symptoms are suggestive of the diagnosis of NP-C. Even with apparently negative Filipin test results, a molecular genetics study is considered essential for prenatal diagnosis for couples with a previous affected mutation-identified child. This test uses chorionic villus sampling or amniotic cells at 10–12 weeks [2, 5, 23]. (3) Nonenzymatic plasma levels of certain cholesterol oxidation products (oxysterols) are biomarkers for clinical screening of NP-C. This is correlated with the age of disease onset and disease severity and may help in monitoring response to treatment [8, 24, 25]. (4) Elevated chitotriosidase activity is known as a marker in Gaucher disease and can be useful in NP-C. It has a relatively low sensitivity and specificity and is more elevated in very young patients [4, 26].

## 5. Management and Treatment

Until recently, there was no curative treatment for NP-C; management remains mainly supportive and symptomatic treatment to improve health-related quality of life (HR-QOL), following the disease-specific treatment. The aims of the treatment are to stabilize or slow disease progression (Table 5).

### 5.1. Symptomatic Management

Gastrointestinal symptoms [2, 4]: (1) patients with dysphagia become malnourished and are at risk of silent aspiration, requiring a softening and thickening of food and antibiotics to avoid aspiration pneumonia. Some patients need gastrostomy to maintain daily caloric intake. (2) Heavy drooling of saliva can be controlled by small doses of oral atropine. (3) Diarrhea associated with miglustat therapy can be managed with antidiarrheal and diet modification.

Neurological symptoms/manifestations [2, 4, 27]: (1) seizures poorly or partially respond to antiepileptic drugs, although increasing drug doses or combining drugs may control severe convulsion. (2) Cataplexy can usually be controlled by tricyclic antidepressants or CNS stimulants. (3) Dystonia and tremors respond well to anticholinergic drugs at least transiently, in some patients. Other effective drugs include trihexyphenidyl and benzodiazepines, and in severe forms of dystonia Gamma-aminobutyric acid derivatives may help. (4) Insomnia, can be treated by Melatonin and sleep apnea by positive airway pressure. (5) In spasticity, physiotherapy is useful and can help in the prevention of contractures. (6) Behavioral and speech problems or schooling difficulties should be referred to psychiatric team and special schooling.

### 5.2. Disease-Specific Treatment

*Miglustat* (N-butyldeoxynojirimycin; NB- DNJ; Zavesca, Actelion pharmaceutical Ltd.) is the only disease-specific oral therapy approved to treat progressive neurological manifestation of NP-C. A small water-soluble, iminosugar molecule reversibly inhibits glycosphingolipid synthesis. It has beneficial effects on lipid trafficking defects, reducing the neurotoxic accumulation of glucosylceramide, lactosylceramide, and gangliosides GM2 and GM3 in the brain and delaying the progression of neurological symptoms of NP-C but it has no effect on the systemic manifestations. In order to stabilize or slow neurological progression, miglustat should be started immediately at the earliest sign of neurological manifestation [2, 4–6, 8, 28, 29]. The recommended dose of miglustat for the treatment of patients 12 years of age and above is 200 mg three times a day and dosing in patients under the age of 12 years should be adjusted based on body surface area [5, 30]. The most common adverse effects associated with miglustat treatment are gastrointestinal with mild to moderate diarrhea, vomiting, flatulence, and weight loss tending to decrease over time. These can usually be managed with antipropulsive medical products such as loperamide. It is also helpful to take miglustat between meals, have a temporary dose reduction, and be on low lactose, sucrose, and maltose diet. Other reported side effects are tremors and mild reduction in platelet counts without associated bleeding tendency [30, 31].

*Tissue and Organ Transplantation.* Bone marrow transplantation or liver transplantation may also be effective, although there is a regression of hepatosplenomegaly and lung infiltration. Transplantation is not effective in treating neurological symptoms in* NPC1* patients. Encouraging results have been reported supporting early hematopoietic stem cell transplantation in* NPC2* patients [2, 5, 32].

*Cholesterol-Lowering Agents.* A combination of low cholesterol diet and hypocholesterolemic drugs may partially reduce the cholesterol load in the liver but does not improve the neurological manifestations [33].

## 6. Treatment Aims and Disease Monitoring

Miglustat was approved in the EU in 2009 and later in non-EU countries for the treatment of progressive neurological symptoms in patients with NP-C [34]. The approval was based on the results of the observational clinical long-term studies. The findings have confirmed the therapeutic effects of miglustat in the stabilization of neurological manifestations. In order to monitor disease progression and patient response to treatment, disease-specific disability scales have been formulated representing the functional disability by semiannual evaluating four key parameters: ambulation, manipulation, language, and swallowing. Evaluation is performed by using a standardized video recording of a clinical examination assessing speech, walking, dysmetria, writing, dystonia, dysphagia, and saccadic eye movements. The video provides useful long-term follow up information. The psychometric evaluation scales by Mini-Mental state examination are widely used in children. Developmental scales can be used in the assessments of infants and children. Other helpful measures are physical examination, hearing evaluation, laboratory nonspecific plasma analysis of liver transaminase, blood counts, annual abdominal ultrasound, and brain magnetic resonance imaging (MRI) [2, 5, 35]. Data analysis of baseline disease characteristics and safety and efficacy of miglustat therapy comes from a prospective clinical trial of 12 children who were enrolled in a pediatric study; patients aged 4 to 12 years received miglustat for up to 24 months and long-term safety and tolerability data from extension therapy up to 52 months. The most frequent manifestations were VSGP (100%), ataxia (83%), splenomegaly (83%) cognitive impairment (67%), dysarthria (58%), hepatomegaly (58%), dysphagia (33%), and cataplexy (33%) [29]. Ten children completed 24 months treatment, miglustat stabilized neurological disease progression, and the key disease parameters (horizontal saccadic eye movement, swallowing, and ambulation) were stabilized at 24 months in 8 of 10 patients (80%). The majority of adverse events were mild to moderate in severity, diarrhea was the most common (67%) gastrointestinal adverse event, and tremor was most common (58%) neurological adverse event. Similar safety and tolerability were observed in juveniles and adults with NP-C disease [29, 36]. The international NP-C registry was initiated in September 2009; as of March 2012, 163 patients from 14 European countries, Australia, Brazil, and Canada have been enrolled in the registry, regardless of their treatment status; 72% were recorded as being treated with miglustat therapy. The median duration of exposure to miglustat was 1.13 year (range 0–7.73 years). Approximately two-thirds of patients had infantile or juvenile onset of neurological manifestations. The most frequent neurological manifestations among all those with available data (*N* = 138) were ataxia (70%), VSGP (70%), dysarthria (66%), cognitive impairment (62%), dysphagia (52%), dystonia (46%), seizures (33%), and cataplexy (26%). The most frequencies of systemic manifestations were neonatal jaundice during infancy (49/126; 39%); hepatomegaly during infancy (50/127; 39%); splenomegaly (69/127; 54%). The prevalence of neonatal jaundice, hepatomegaly, and/or splenomegaly during infancy was greatest among early infantile onset patients and decreased with increasing age at neurological onset, compared with 21% of adolescent/adult-onset patients who had a history of neonatal jaundice and 30% who had hepatosplenomegaly during infancy [14]. Patient disability was assessed using a disability scale with the four domains (ambulation, manipulation, language, and swallowing ability), and scores were rated from 0 (normal) to 1 (worst); patients were categorized as improved/stable if ≥3 out of 4 domain scores were lower or unchanged, or as progressed if <3. As of October 2013, 283 patients from 19 countries were enrolled in the registry, of which 92 (33%) had received ≥12 months continuous miglustat therapy with mean (range) of 2.0 (1.0–3.7) years. Of 84 evaluated patients, 9 (11%) had early infantile onset, 27 (32%) had late infantile onset, 30 (36%) had juvenile onset, and 21% had adolescent/adult onset of neurological manifestations. Overall, 69% of continuously treated patients with available data were categorized as “improved/stable”: 33% early infantile patients; 50% late infantile patients; 79% juvenile patients; 94% adolescent/adult patients and 31% patients were categorized as progressed. Based on that analysis, it was revealed that the proportion of patients categorized as “improved/stable” increased with increased age at onset of neurological presentations [37]. An observational study (median age at diagnosis, 8 years) examined therapeutic outcomes in German cohort of patients with NP-C who had received miglustat for ≥2 years; the treatment slowed the disability progression, with the most marked benefit seen in juvenile onset; initiation of miglustat early in patients with only slight or moderate neurological symptoms may support better therapeutic outcomes [38].

## 7. Prognosis

Correlating with age at onset of neurological signs, patients with an early onset form of NP-C progress faster and death occurs most often between 3 and 5 years of age. Those with a late infantile neurologic onset usually die between 7 and 12 years. Patients with fetal hydrops usually die within a few days after birth. The systemic disease can be fatal in early infancy due to hepatic or respiratory failure, death occurring before 3–6 months of age. Otherwise, unexplained neonatal cholestatic jaundice is transient and usually resolves spontaneously by four months of age. Splenomegaly very rarely leads to pancytopenia. Dysphagia associated with aspiration pneumonia is a major cause of mortality. Severe and intractable epilepsy is a risk factor for poor prognosis [2, 4, 39]. The treatment with miglustat of patients with perinatal and infantile forms (patients who generally have symptoms more severe and rapid disease progression) may be less effective than those with other forms. For patients whose diagnosis is based on sibling screening or systemic manifestation of NP-C with initiation of miglustat at the first neurological sign, the response is generally better. There is very limited data of effective of treatment with miglustat to prevent neurological symptoms for asymptomatic patients [4, 8].

## 8. New Ongoing Therapies in Development Based on Experimental Data

(1) Histone deacetylase inhibitors (HDACi) for stabilizing NPC1 missense mutant proteins, reducing cholesterol accumulation, and preventing the rapid degradation of mutant NPC1 proteins [40]: a first-in-human proof-of-concept Phase I trial is recruiting mid-2014 [41].

(2) Sterol-binding agents and early treatment with 2-hydroxy-beta-cyclodextrin (2-HP-beta-CD) have been reported to reduce both cholesterol and sphingolipids storage and have led to prolonged survival in* NPC1* in animal models. A Phase I clinical trial in 2013 for intrathecal HP-beta-CD is underway [41, 42].

## 9. New Biomarkers for the Diagnosis or for Assessing Efficacy of Treatment in Patients with NP-C

(1) Urinary bile acids of 3 beta-sulfooxy-7 beta-N-acetylglucosaminyl-5-cholen-24-oic acid (SNAG-Δ^5^-CA) and the glycine and taurine-conjugates (SNAG-Δ^5^-CG and SNAG-Δ^5^-CT) appeared to be elevated in patients with NP-C [43, 44].

(2) Plasma lysosphingolipids, lysosphingomyelin (SPC), and glucosylsphingosine (GlcSph) are reported to be high in patients with NP-C. The SPC is a sensitive and specific diagnostic marker while GlcSph is elevated in a subset of NP-C patients [45, 46].

(3) The ratios of choline (Cho) and N-acetyl aspartate (NAA) or (Cho) and creatine can be used as markers of neuronal loss and dysfunction using brain proton magnetic resonance spectroscopy (H-MRS). Increases in these ratios have been shown in a number of diseases including* NPC1*. A decrease in the Cho/Cr ratio has previously been noted in patients with miglustat therapy as an assessment of treatment response [47, 48].

## 10. Conclusion

The Niemann-Pick NP-C disease characterized by highly heterogeneous presentation, making for difficult and often delayed diagnosis; The NP-C Suspicion Index (SI) is a screening tool offer considerable help in the diagnostic process of patients with suspicion of NP-C. The SI comprises ranked assessments to provide an NP-C Risk Predication Score (RPS). A RPS is calculated by the presence of clinical manifestations of NP-C within and across three domains (visceral, neurological, and psychiatric) plus family history information. The NP-C SI tool has strong predictive power for suspicion of NP-C in patients >4 years of age but it is less able to discriminate NP-C in infants <4 years of age. A more reliable tool for use in the pediatric population is needed. The definitive diagnosis requires Filipin staining of cultured skin fibroblasts and genetic sequencing of* NPC1* and* NPC2* mutations. New diagnostic biomarkers are being made for rapid diagnosis of NP-C. Miglustat is the first disease-specific approved therapy for the treatment of neurological manifestations and should be initiated at the earliest signs of neurological manifestations, in order to stabilize or slow the irreversible neurological damage. New therapies are underway. There is a need for raising disease awareness and improving early detection, for optimal disease management.

## Figures and Tables

**Table 1:** Common differential diagnosis to the presenting symptoms of the NP-C at different age groups (2–6).

Age group	Presenting symptoms	Common differential diagnosis
Pre-/perinatal period (<3 mo)	Fetal hydrops	Chromosomal disorders Congenital heart malformations Hemoglobinopathies Infectious diseases
	Prolonged neonatal cholestatic jaundice	Idiopathic neonatal hepatitis Biliary atresia, galactosemia Alpha 1 antitrypsin deficiency Bile acid synthesis disorders Cystic fibrosis, tyrosinemia type I Byler disease Peroxisomal disorders

Early infantile period (3 mon to <2 yrs) and late infantile period (2 to <6 yrs)	Isolated splenomegaly or hepatosplenomegaly	Mucopolysaccharidosis Oligosaccharidosis, sphingolipidosis (Gaucher disease, Niemann-Pick A and B) Lipid storage disease (Wolman) Glycogen storage disorders

Late infantile period and juvenile period (6–15 yrs)	Dystonia	Respiratory chain disorders Pyruvate dehydrogenase deficiency, vitamin E deficiency Glucose transporter 1 deficiency Homocystinuria, Wilson disease Urea cycle defects Organic aciduria
	Ataxia	Mitochondrial disorders Friedreich's ataxia, vitamin E deficiency, and autosomal recessive cerebellar ataxia (ARCA)
	Vertical supranuclear gaze palsy (VSGP)	Progressive supranuclear palsy Multiple system atrophy Dementia with Lewy bodies Spinocerebellar ataxia, Tay-Sachs disease, Wilson disease, and vitamin B12 deficiency Wernicke encephalopathy Huntington's disease Creutzfeldt Jakob disease
	Gelastic cataplexy	Gelastic seizures Narcoleptic tetrad

Juvenile period (6–15 yrs)	Psychosis	Hysteria, schizophrenia, and Wilson disease Urea cycle defect Acute intermittent porphyria Cerebrotendinous xanthomatosis, homocystinuria

**Table 2:** Scoring of manifestations, the combination of symptoms, and the patient's family history, to provide the total NP-C Risk Predication Score (RPS) [4, 9].

Very strong symptoms (40 points per item)	Strong symptoms (20 points per item)	Moderate symptoms (10 points per item)	Weak symptoms (5 points per item)	Ancillary symptoms (1 point per item)
Vertical supranuclear gaze palsy (VSGP) [N]	Isolated splenomegaly with or without hepatomegaly [V]	Ataxia clumsiness or frequent falls [N]	Acquired and progressive spasticity [N]	(i) Hydrops fetalis [V] (ii) Sibling with fetal ascites [V]

Gelastic cataplexy [N]	Prolonged neonatal cholestatic jaundice [V]	Dysarthria and/or dysphagia [N]	Treatment-resistant psychiatric symptoms [P]	Delayed developmental milestones [N]

	Cognitive decline [P]	Dystonia [N]	Other psychiatric disorders [P]	Seizures [N]

		Psychotic symptoms [P]		Disruptive or aggressive behavior in adolescence and childhood [P]

				(i) Hypotonia [N] (ii) Myoclonus [N]

(i) N: neurological domain symptoms, V: visceral domain symptoms, and P: psychiatric domain symptoms.

(ii) Combined manifestations (scores): V + N = 40 points, V + P = 40 points, and P + N = 20 points.

(iii) Family history (scores): 1st degree = 40 points and 2nd degree = 10 points.

(iv) Total Risk Predication Score for NP-C: <40 = low probability of NP-C and alternative causes should be considered. A score of 40–69 = moderate suspicion of having NP-C; further follow-up observation is required and contact NP-C referral center for further discussion. A score of ≥70 = high suspicion of having NP-C and should be immediately referred to an NP-C center for testing for NP-C.

**Table 3:** Manifestations of NP-C disease by age group [10].

Age <4 years	Age 4–16 years	Age >16 years
Splenomegaly (>50%) Prolonged neonatal unexplained or cholestasis jaundice (>50%) Delay of the developmental milestones (>50%) Infantile hypotonia Dystonia Dysarthria/dysphagia	Dystonia Vertical supranuclear gaze palsy Dysarthria/dysphagia Seizure, delayed development milestones, gelastic cataplexy, and ataxia Cognitive decline Splenomegaly	Vertical supranuclear gaze palsy Dystonia, dysarthria/dysphagia Ataxia Cognitive decline Psychotic symptoms, treatment-resistant psychiatric symptoms, disruptive or aggressive behavior, and other psychiatric disorders

Majority of domain symptoms Visceral + neurological	Majority of domain symptoms Neurological + psychiatric. Visceral + neurological Visceral + psychiatric	Majority of domain symptoms Psychiatric + neurological

**Table 4:** Association of manifestations by the presence and absence of leading NP-C manifestations (ataxia, cognitive decline, psychosis, and splenomegaly) at age >4 years [10].

With ataxia (a moderate indicator)	With psychosis (a moderate indicator)	With cognitive decline (a strong indicator)	With splenomegaly (a strong indicator)
Dystonia, dysarthria/dysphagia Cognitive decline (i) Patients without ataxia show increase frequency of VSGP and cognitive decline	Treatment-resistant psychiatric symptoms Dysarthria/dysphagia Dystonia (i) Patient presentation without psychosis shows increase in frequency of VSGP, cognitive decline, dysarthria/dysphagia, and ataxia	Psychotic symptoms Treatment-resistant psychiatric symptoms (slightly elevated) Ataxia Gelastic cataplexy Dystonia and seizures (i) The patients without symptoms of cognitive decline show increased frequency of other types of psychiatric disorders	There is little difference (i) Patient's presentation without splenomegaly shows a slight increase in tendency of prolonged neonatal jaundice, ataxia, and dysarthria/dysphagia

VSGP: vertical supranuclear gaze palsy.

**Table 5:** Summary of treatment strategy in NP-C patients.

	Type of treatment
(1) Symptomatic treatment	
(a) Gastrointestinal sign	
(i) Dysphagia	(i) Diet softening and thickening (ii) Gastrostomy
(ii) Heavy drooling of saliva	(i) Oral atropine
(iii) Diarrhea with Miglustat	(i) Antidiarrheal agent and reducing dietary sucrose, maltose, and lactose
(b) Neurological sign	
(i) Seizures	(i) Antiepileptic drugs
(ii) Cataplexy	(i) Tricyclic antidepressants or CNS stimulants
(iii) Dystonia	(i) Anticholinergic drugs Trihexyphenydil, benzodiazepines, and Gamma-aminobutyric acid
(iv) Insomnia	(i) Melatonin
(v) Sleep apnea	(i) Positive airway pressure
(vi) Spasticity	(i) Physiotherapy
(vii) Behavioral or speech problems or schooling difficulties	(i) Referring to psychiatric team and special schooling

(2) Disease-specific treatment	(i) Miglustat (Zavesca) (ii) Tissue and organ transplantation (bone morrow, liver, and hematopoietic stem cells) (iii) Cholesterol-lowering agents

(3) New ongoing treatment	(i) Histone deacetylase inhibitors (ii) 2-Hydroxy-*β*-cyclodextrin

## List of Abbreviations

NP-C: Niemann-Pick type C
*NPC1*: Niemann-Pick type C with mutations in the* NPC1* gene
*NPC2*: Niemann-Pick type C with mutations in the* NPC2* gene
SI: Suspicion Index
VSGP: Vertical supranuclear gaze palsy
SEM: Saccadic eye movement
VSEM: Vertical saccadic eye movement
HSEM: Horizontal saccadic eye movement
EEG: Electroencephalogram
MRI: Magnetic resonance imaging
MRS: Magnetic resonance spectroscopy
NP-C SI: NP-C Suspicion Index
RPS: Risk prediction score
ROC: Receiver operating characteristic curve
AUC: Area under the curve.
